# The Fast and Easy Way for Double-Lumen Tube Intubation: Individual Angle-Modification

**DOI:** 10.1371/journal.pone.0161434

**Published:** 2016-08-18

**Authors:** Jeong Jin Min, Jong-Hwan Lee, Se Hee Kang, Eunhee Kim, Sangmin M. Lee, Jong Ho Cho, Hong Kwan Kim

**Affiliations:** 1 Department of Anesthesiology and Pain Medicine, Samsung Medical Center, Sungkyunkwan University School of Medicine, Seoul, Korea; 2 Department of Thoracic and Cardiovascular Surgery, Samsung Medical Center, Sungkyunkwan University School of Medicine, Seoul, Korea; University of Colorado Denver, UNITED STATES

## Abstract

To find the faster and easier way than the existing intubating technique for double-lumen tube, we modified the angle of double-lumen tube according to an individual’s upper airway anatomy and compared the time needed and the number of attempts for successful intubation between individually angle-modified and non-modified double-lumen tubes. Adult patients undergoing elective thoracic surgery were randomly allocated in either non-angle-modified (Group N, n = 54) or angle-modified (Group M, n = 54) groups. During mask ventilation in the sniffing position, angle-modification was performed in Group M as follows: the distal tip of the tube was placed at the level of the cricoid cartilage and the shaft was bent at the intersection of the oral and pharyngeal axes estimated from the patient’s surface anatomy. The time needed and the number of attempts for successful intubation and Cormack and Lehane (C-L) grade were recorded. Overall median intubation time (sec) was significantly shorter in Group M than in Group N [10.2 *vs*. 15.1, *P*<0.001]. In addition, Group M showed the shorter median intubation time (sec) in C-L grades I-III [8.2 *vs*. 11.1 in C-L grade I, (*P* = 0.003), 10.3 *vs*. 15.3 in II, (*P* = 0.001), and 11.8 vs. 27.9 in III, (P<0.001), respectively]. Moreover, all intubation was successfully performed at the first attempt in patients with C-L grades I-III in Group M (*P* = 0.027). Our study showed an individual angle-modification would be useful for the fast and easy intubation of double-lumen tube in patients with C-L grades I-III.

***Trial Registration*:** ClinicalTrials.gov NCT02190032

## Introduction

A double-lumen tube is more difficult to insert than a single-lumen tube mainly because of its wider external diameter, less compliant characteristics, and straighter shape, [1–3] although it has been generally accepted as a standard technique for lung isolation during thoracic surgery. [1] Moreover, various videolaryngoscopic devices, in spite of the successful achievement of better laryngeal views, have failed to show the superiority to the direct laryngoscopy for the faster placement of double-lumen tube. [3–5]

Basically, tracheal intubation is composed of three sequential steps: 1) the achievement of laryngeal view, 2) the delivery of tube to the glottis, and 3) the advancement of tube into trachea. [6, 7] Therefore, the ability to visualize the larynx might not be sufficient for the fast and successful intubation of a double-lumen tube. Moreover, considering the distinguishing characteristics of double-lumen tube, accurate delivery of the tube to the glottis might also be a crucial step, although a good laryngeal view is a common important step for tracheal intubation. [8–10] Theoretically, modification of the tube shape according to each patient’s upper airway axes might facilitate tube delivery to the glottis and make inserting a double-lumen tube easier and faster. However, to our knowledge, the usefulness of angle-modification has never been studied for inserting double-lumen tube.

Therefore, we hypothesized that the individually angle-modified double-lumen tube is superior to the manufacturer-provided double-lumen tube with respect to the time and the number of attempts needed for successful intubation. The aim of this study was to evaluate the usefulness of individual angle-modification in patients requiring double-lumen tube intubation.

## Methods

This prospective, randomized, single-blind study was approved by the Institutional Review Board of Samsung Medical Center (SMC 2014-06-003-004) and was registered at ClinicalTrials.gov (NCT02190032). We obtained written informed consent from each participant prior to the study.

### Patients and Randomization

**Fig 1:**
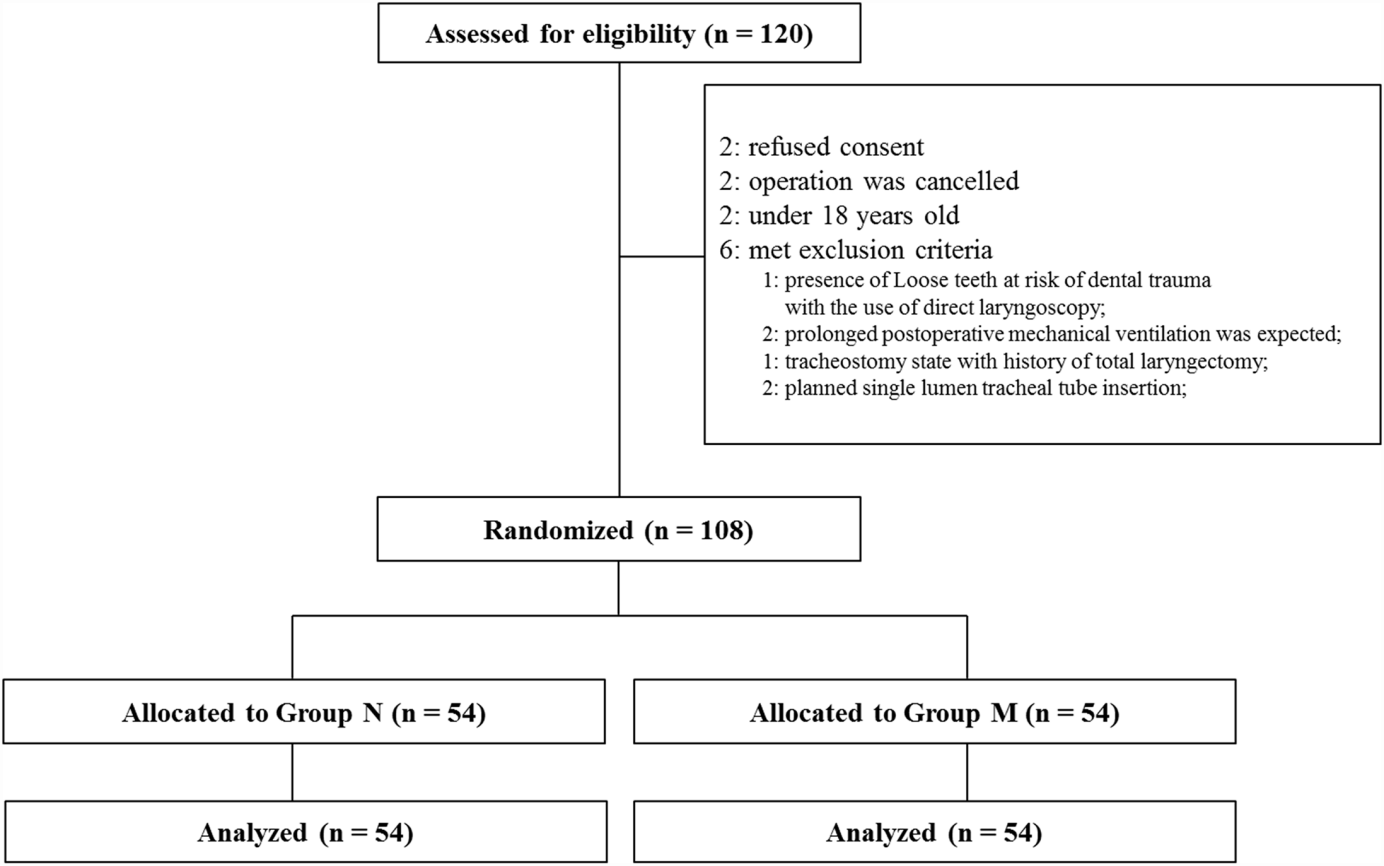
Consort diagram. Group N, non-modified double-lumen tube group; Group M, individually angle-modified double-lumen tube group

We enrolled 108 adult patients who required double-lumen tube insertion for elective thoracic surgery from July 2014 to January 2015. Exclusion criteria were as follows: cervical spine disease that restricted head extension, such as rheumatoid arthritis with atlantoaxial subluxation or cervical disc disease, oropharyngeal obstructive disease; patients requiring rapid sequence intubation; presence of loose or vulnerable tooth; and pregnancy. However, patients were not excluded simply because they had previous history of known difficult airways. Patients were randomly allocated by an independent anesthesiologist (E. H. Kim) into either a non-modified double-lumen tube group (group N, n = 54) or an individually angle-modified double-lumen tube group (group M, n = 54) according to the tube angle using a computer-generated random number table (Fig 1).

### Preoperative airway evaluation

During the preoperative visit, the airway was assessed by examining mouth opening, modified Mallampati score, and thyromental distance. Mouth opening was defined as the distance between the upper and lower incisors or gingiva in edentulous patients and was measured in centimeters with the mouth fully opened. Mallampati classification was determined with the patient in a sitting position with the mouth fully open and tongue protruding without phonation. [11] Thyromental distance was measured along a straight line from the thyroid notch to the lower border of the mandibular mentum with the head fully extended.

### Tube angle modification

The 37- and 35-Fr left-sided double-lumen tubes (Broncho-Cath, Mallinckrodt Medical Ltd., Athlone, Ireland) were used in male and female patients, respectively. Patients in group N were intubated with non-modified, manufacturer-provided double-lumen tubes, and patients in group M were intubated with individually angle-modified double-lumen tubes.

**Fig 2:**
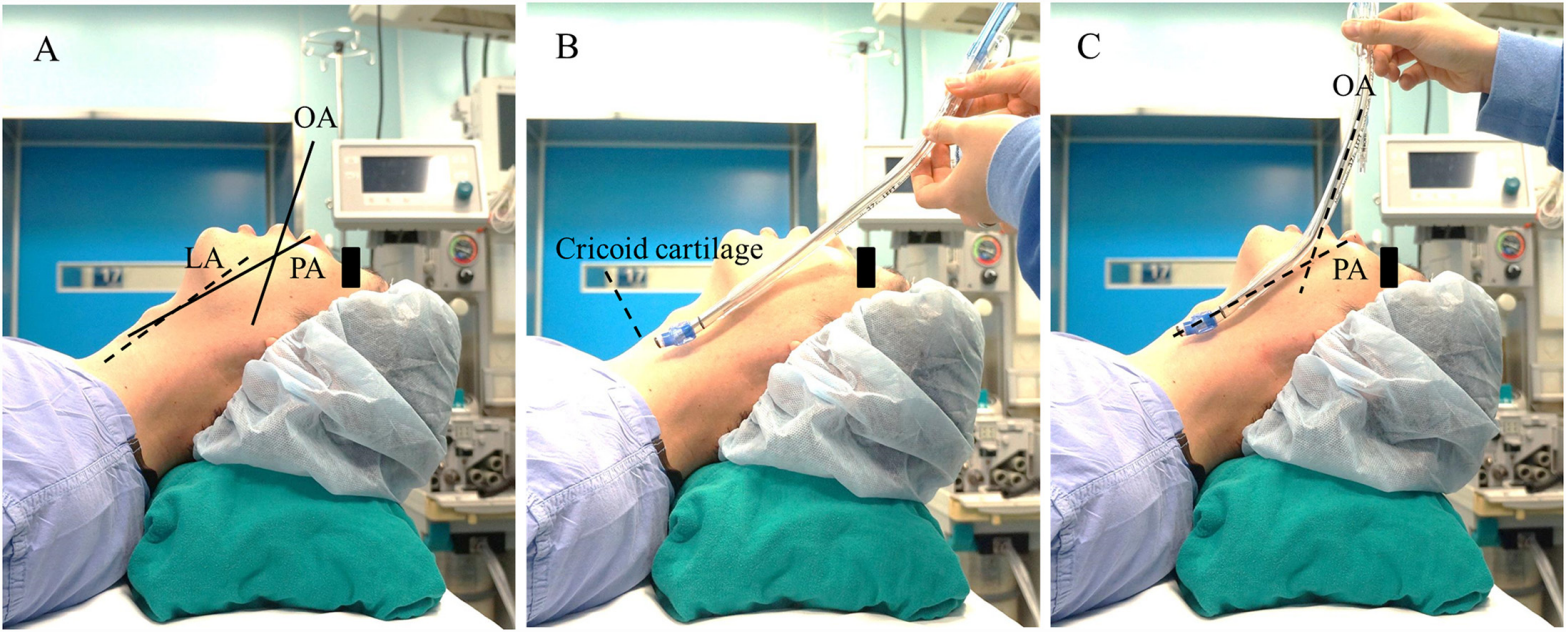
Tube angle-modification in the angle-modified double-lumen tube group. OA, oral axis; PA, pharyngeal axis; (A) With the patient’s head in the sniffing position, the oral and pharyngeal axes were assessed on the lateral side of the face. (B) The distal tip of the tube was positioned at the upper margin of the cricoid cartilage. (C) The tube shaft was bent at the intersection of the imaginary oral and the pharyngeal axes in order to produce a fluent curve.

In group M, the tube was modified in the sniffing position by the same anesthesiologist (E. H. Kim) who assigned the groups as following steps. First, each airway axes was estimated on the lateral side of an patient as follows: oral axis was estimated as a straight line drawn from the tip of the upper incisors parallel to the hard palate; laryngeal axis was estimated as a straight line from the cricoid cartilage near-parallel to a patient’s anterior neck line; pharyngeal axis was estimated as a straight line drawn from the upper margin of cricoid cartilage with an angle of 10–15° posteriorly to the laryngeal axis; [12] (Fig 2A). Second, the distal tip of tube was placed at the upper margin of the cricoid cartilage (Fig 2B). Third, in order to create a fluent curve, the tube was bent at the intersection between the estimated oral and pharyngeal axes while maintaining the alignment of upper and lower parts of tube with the estimated oral and pharyngeal axes, respectively (Fig 2C). During the tube modification, a patient’s mouth was slightly opened passively. All three steps for the tube modification were implemented during the mask ventilation and took no more than 15 seconds.

### Anesthesia and Intubation

Anesthetic drugs and techniques were standardized in all patients. Each patient was laid on the operating table with a 7-cm-high cushion under the head. Non-invasive blood pressure, three-lead ECG, and oxygen saturation were monitored. Anesthesia was induced with intravenous thiopental sodium (5 mg/kg) and continuous infusion of remifentanil (0.2–0.3 mcg/kg/min). Neuromuscular blockade was obtained with intravenous rocuronium bromide (0.6 mg/kg). Lungs were ventilated via a face mask with 5 vol% sevoflurane. Sufficient neuromuscular blockade was assessed by Train-of-Four monitoring. In the sniffing position, patients were intubated with a double-lumen tube according to group using a Macintoch 3 or 4 laryngoscope blade. Once the stylet in the bronchial lumen was removed when the tip of the tube was past the glottis, the tube was rotated 90° counterclockwise in both groups.

One of two experienced anesthesiologists (J. J. Min and J.-H. Lee), not aware of the exact procedure, remained outside the operating room until the tube modification was completed and then performed all intubations. Intubation data including intubation attempts, application of the BURP (backward, upward, and rightward pressure on the larynx) maneuver, [13] and any oropharyngeal injuries were recorded by an independent anesthesiologist (S. H. Kang). The best laryngeal view obtained with or without BURP was recorded by an anesthesiologist performing the intubation (J. J. Min or J.-H. Lee) according to the Cormack and Lehane (C-L) grading scale. [14] The intubation difficulty scale score was also calculated. [15]

### Intubation time and protocol

The intubation time was defined as the length of time from the passage of laryngoscope through the patient’s lip to the passage of double-lumen tube through the vocal cords, which was announced verbally by the anesthesiologist performing the intubation. [4] Each intubation attempt took no more than 60 seconds. If the first intubation attempt with the allocated angled tube failed within 60 seconds, we discontinued the intubation and ventilated the patient with a mask for several breaths. Then, one more attempt with the allocated angled tube was permitted. If the second attempt failed within 60 seconds, the patient was intubated using an appropriate alternative intubation device.

### Postoperative complications

Postoperative sore throat or hoarseness was evaluated in the postoperative 30 minutes and 24 hours, respectively, by an independent observer (H. K. Kim or J. H. Cho) who was unaware of the study groups. Each complication was scored by the patient using a numerical rating scale from 1 to 3, with 1 indicating mild, 2 as moderate, and 3 as severe.

### Statistical analysis

The primary outcome was intubation time. Assuming a meaningful difference of 10 sec in mean intubation time between the two groups with a standard deviation (SD) of 15 sec, sample size was determined with a power of 0.9 and a type 1 error of 0.05. Power analysis suggested that a minimum of 49 patients per group was required. Considering a dropout rate of 10%, we enrolled 54 patients per group.

Data were presented as mean (SD), median [interquartile range], or numbers of patients (%), as appropriate. Normality of the data distribution was determined with a Kolmogorov-Smirnov test. Student’s *t*-test or Mann-Whitney *U*-test was used to compare the continuous variables between the groups as appropriate. For subgroup analyses, *P* values were adjusted using Bonferroni correction. The Chi-square or Fisher’s exact test was used to compare the frequencies of categorical variables. Pearson’s correlation was used to analyze the relationship between C-L classification and the number of attempts until successful intubation. All data were analyzed using SPSS version 20.0 software (IBM Corp., Armonk, NY, USA).

## Results

Of the 120 patients who were screened for eligibility, 12 were excluded because they did not meet inclusion criteria or refused to participate in the study (Fig 1). A total of 108 patients between 20 and 75 years of age completed the study (n = 54 in each group). There were no differences in baseline characteristics between the two groups (Table 1).

**Table 1:** Baseline characteristics and Airway Assessment. Data are presented as mean (SD) or number (%). No significant difference in any of those variables was observed between the two groups.

	Group N (n = 54)	Group M (n = 54)
Age (years)	54.9 (12.8)	52.7 (12.6)
Male (%)	30 (56%)	32 (59%)
Height (cm)	163.9 (8.7)	164.2 (11.2)
Weight (kg)	62.9 (12.4)	63.9 (14.3)
BMI (kg/cm^2^)	23.3 (3.6)	23.6 (3.8)
Mallampati class (I/II/III/IV)	22/27/5/0	24/26/4/0
Inter-incisor distance (cm)	4.3 (0.5)	4.5 (0.5)
Passive mouth opening (cm)	3 (0.7)	3.1 (0.6)
Thyromental distance (cm)	6.5 (0.6)	6.5 (0.7)
C-L grade without BURP (I/II/III/IV)	23/19/12/0	19/21/13/1
C-L grade with BURP (I/II/III/IV)	35/16/3/0	21/26/6/1

Group N, non-modified double-lumen tube group; Group M, individually angle-modified double-lumen tube group; BMI, body mass index; C-L, Cormack and Lehane; BURP, backward, upward, and rightward pressure on the larynx

Among 108 enrolled patients, 101 (94%) were successfully intubated on the first attempt. Among the seven patients who required more than one intubation attempt, six were in group N and one was in group M (Table 2). All five patients in group N who failed intubation after two attempts were successfully intubated on the third attempt with angle modification of the double-lumen tube, according to our study method. Detailed data of the seven patients who failed to be intubated on the first attempt are presented in Table 3. Although the total success rate of tracheal intubation with an allocated double-lumen tube within two attempts did not differ between the groups (*P* = 0.2), initial intubation success rate was significantly higher in group M compared to group N in 107 patients with C-L grade I-III (*P* = 0.027) (Table 2).

**Table 2:** Intubation Outcome Data.

	Group N	Group M	*P* value
**Overall**			
Number of patients, n	54	54	
Intubation Success, n (%)			
First attempt	48 (89%)	53 (98%)	0.11
Second attempt	49 (91%)	53 (98%)	0.21
Intubation time (sec)	15.06 [11.01–25.44]	10.20 [7.80–14.55]	< 0.001*
Need for BURP	29 (54%)	18 (33%)	0.033*
IDS score	1 [0–2.25]	1 [0–2]	0.606
IDS score > 5, n (%)	5 (9%)	1 (2%)	0.21
**Subgroup analysis of patients with C-L grade I-III**			
Number of patients, n	54	53	
Intubation Success, n (%)			
First attempt	48 (89%)	53 (100%)	0.027*
Intubation time (sec)			
C-L grade I	11.09 [8.43–12.95]	8.16 [6.30–9.97]	0.003*
I	15.25 [13.33–22.10]	10.30 [8.01–15.43]	0.001*
III	27.90 [25.44–120]	11.84 [10.75–20.49]	<0.001*
C-L grade with BURP			
I	12.95 [10.26–18.65]	8.18 [6.79–10.85]	0.001*
II	25.48 [15.00–52.04]	10.94 [8.48–15.93]	0.001*
III	120 [13.81–120]	10.76 [9.49–13.63]	0.038

Data are presented as median [interquartile range] or number (%).

*P < 0.05 *vs*. Group N. Mann-Whitney *U*-test was used to compare the continuous variables between the groups and the Chi-square or Fisher’s exact test was used to compare the frequencies of categorical variables. Group N, non-modified double-lumen tube group; Group M, individually angle-modified double-lumen tube group; C-L, Cormack and Lehane; BURP, backward, upward, and rightward pressure on the larynx; IDS, intubation difficulty scale.

**Table 3:** Detailed Description of the Patients Who Failed the First Intubation Attempt.

Group	Sex/Age	BMI	MPT	C-L	C-L-B	No. of attempts	Comments
1: N	M/52	22.8	1	3	2	2	Successful intubation on the 2nd attempt with the allocated tube (non-modified tube) in 32.27s
2: N	F/43	20	2	2	1	3	Successful tracheal intubation after tube angle modification in 25s on 3rd attempt
3: N	M/58	16.1	2	3	2	3	Successful tracheal intubation after tube angle modification in 13.26s on 3rd attempt
4: N	F/47	27.2	1	3	3	3	Successful tracheal intubation after tube angle modification in 10.7s on 3rd attempt
5: N	M/57	25.8	2	3	3	3	Successful tracheal intubation after tube angle modification in 11.97s on 3rd attempt
6: N	M/42	27.6	2	3	2	3	Successful tracheal intubation after tube angle modification in 23.8s on 3rd attempt
7: M	M/68	21.8	3	4	4	5	Small mouth open, short TMD, and C-L grade IV with or without BURP technique;
							Single-lumen endotracheal tube inserted using light wand and subsequent one lung ventilation was achieved with a bronchial blocker

Group N, non-modified double-lumen tube group; Group M, individually angle-modified double-lumen tube group; BMI, body mass index; MPT, Mallapati classification; C-L, Classic Cormack and Lehane classification; C-L-B, Cormack and Lehane grade with BURP (backward, upward, and rightward pressure on the larynx) technique.

**Fig 3:**
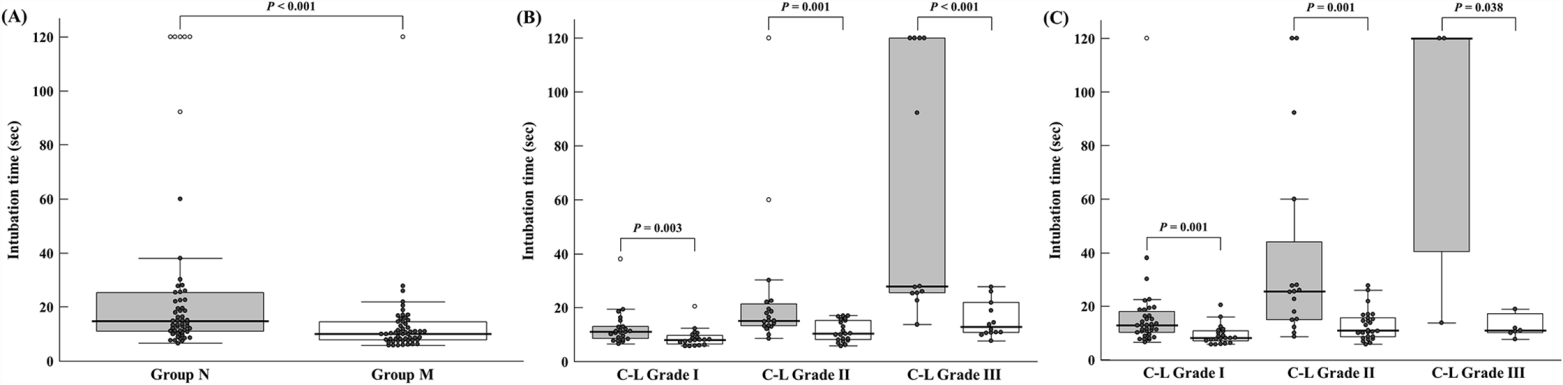
Comparison of the median intubation times between the groups. Group N, non-modified double-lumen tube group; Group M, individually angle-modified double-lumen tube group). (A) Overall analysis of the patients. (B) Subgroup analysis according to the Cormack and Lehane (C-L) grade. (C) Subgroup analysis according to the Cormack and Lehane (C-L) grade with the BURP (backward, upward, and rightward pressure on the larynx) technique.

Overall, the median time for tracheal intubation was significantly shorter in group M compared to group N [median (inter-quartile range): 15.6 (11.0–25.4) vs. 10.2 (7.8–14.6), *P* < 0.001]. In the 101 patients who were successfully intubated on the first attempt, intubation time was also shorter in group M compared to group N [median (IQR): 13.6 (10.7–19.5) vs. 10.1 (7.8–14.2), *P* < 0.001). In subgroup analyses of intubation time according to C-L grade, intubation time was significantly shorter in group M than in group N except for patients with C-L grade III after applying the BURP technique (Table 2, Fig 3).

The distribution of C-L grade with or without BURP technique was not different between the two groups (Table 1). Total number of intubation attempts was significantly associated with C-L grade (*r* = 0.39, *P* < 0.001). External laryngeal manipulation with the BURP technique for tracheal intubation was necessary in 54% of patients in group N and 33% in group M (*P* = 0.03, Table 2). There was no significant difference in intubation difficulty score between the two groups (*P* = 0.606, Table 2).

Postoperative complications, including sore throat and hoarseness, were not significantly different between the two groups (Table 4). Eleven patients (three in group N and eight in group M) were excluded from postoperative complication analysis because they were changed to single-lumen endotracheal tubes at the end of the surgery per surgeon request. Mean operation time (min) was not different between the two groups (189 (110) in group N and 175 (73) in group M, *P* = 0.984).

**Table 4:** Postoperative Complications.

	Group N (n = 51)	Group M (n = 46)	*P* value
0.5 h postoperative			
Sore throat	12 (23.5%)	7 (15.2%)	0.303
Hoarseness	18 (35.3%)	12 (26%)	0.327
24 h postoperative			
Sore throat	6 (11.8%)	3 (6.7%)	0.492
Hoarseness	14 (27.5%)	8 (17.8%)	0.260

Data are presented as number (%). The incidence of postoperative complications was compared using Chi-square (for 0.5 h postoperative sore throat, hoarseness and 24 h postoperative hoarseness) or Fisher’s exact test (for 24 h postoperative sore throat). There were no differences between the two groups. Group N, non-modified double-lumen tube group; Group M, individually angle-modified double-lumen tube group.

## Discussion

In this randomized controlled trial, individually angle-modified double-lumen tubes significantly reduced the time for successful tracheal intubation. In the subgroup analysis, intubation time was also shorter in the angle-modified group than in the control group for all C-L grades except for patients with C-L grade III after applying the BURP technique. In the angle-modified group, patients with C-L grades I-III all had successful tracheal intubations on the first attempt.

Tracheal intubation consists of three challenging sequential steps as previously described by Levitan and colleagues, which include achieving laryngeal view, delivering the tube tip to the glottic opening, and advancing the tube into the trachea. [6] Generally, an easier intubation can be expected with an easier laryngeal view. [16] Several earlier studies with double-lumen tube insertion focused on achieving a better laryngeal view using one of various videolaryngoscopic devices including the GlideScope, Airtraq, or CEL-100. [3–5] However, most of those studies failed to show the superiority of those devices to direct laryngoscopy with regard to the intubation time or related complications.

To explain those interesting findings, we focused the specific characteristics of double-lumen tube. The double-lumen tube’s larger external diameter, longer straight portion, and more rigid structure theoretically would make delivering the tube to the laryngeal inlet more difficult. Therefore, even with the better laryngeal view achieved with the videolaryngoscopic devices, limited airway passage space alongside the devices would limit the ease of tube delivery to the laryngeal inlet. In addition, only a previous double-lumen tracheal intubation study using the relatively non-space-occupying device (OptiScope) and the tube-through-device technique has shown that a rigid video-stylet offered the superior results compared to the Macintosh laryngoscope, including shorter intubation time and less trauma. [17]

Based on those previous results, when using direct laryngoscopy, we hypothesized that the modified double-lumen tube aligned with a patient’s airway axes would allow the uninterrupted passage from the mouth to the vocal cords. Therefore, in this study, the double-lumen tubes were modified according to the imaginary line made by the oral and pharyngeal axes estimated from an individual’s surface landmarks. Because the cricoid cartilage is generally palpable and located slightly below the laryngeal inlet, the modification was started from placing the distal tip of double-lumen tube at the upper margin of cricoid cartilage. Following the tip placement, the tube was bent at the intersection between a patient’s oral and pharyngeal axes to create a fluent oropharyngeal curve resembling the primary curve of airway passage. [18] During the entire procedures, a patient’s mouth was passively opened because intubation with direct laryngoscopy requires the mouth opening. The shapes of modified double-lumen tubes were slightly different from patient to patient depending upon the degree of mouth, thyromental distance, and neck extension. Therefore, we defined our method as individual angle-modification.

In the daily clinical practice, many anesthesiologists bend the double-lumen tube after one scout laryngoscopy. However, repeated laryngoscopy has been known to increase morbidity; therefore, it is recommended that the best intubation conditions be determined for the initial laryngoscopy. [19] In this regard, our method would be the way to achieve the optimal intubating conditions for the double-lumen tube at the first attempt.

In the subgroup analysis according to C-L grade, tube angle modification significantly reduced the median intubation time in all C-L grades except for patients with C-L grade III with BURP technique. The difference in median intubation time between the two groups increased at higher C-L grades (grade II or III) compared to grade I. Although individual angle modification did not shorten intubation time in patients with C-L grade III after applying the BURP technique, there was a considerable difference between groups M and N ([median]: 120 sec *vs*. 10.76 sec). Considering that there were only three patients in group M in this subgroup analysis, it might be hard to conclude the efficacy of angle modification in these patients. Moreover, in six patients with C-L grade II or III and two failed intubation attempts with the conventional tube, the third intubation attempts via tube angle modification according to our study methods were all successful. In the angle-modified group, all patients with C-L grade I-III were successfully intubated on the initial laryngoscopy except for one patient with C-L grade IV.

There were several limitations in our study. First, we did not compare the different angle modification techniques. For single-lumen tube intubation, bending the tube with a ‘straight-to-cuff’ method at an angle less than 35 degrees is generally recommended. [20] Because the manufacturer-provided double-lumen tube is slightly bent at the bronchial cuff level, we regarded the conventional tube as the ‘straight-to-cuff’ designed tube. In another double-lumen tube intubation study by Hsu et al., the tube was angled at the level of the tracheal orifice with or without exposure of the orifice. [21] Therefore, further studies on this issue are needed. Second, we recorded the laryngeal view using the classic C-L classification [14] and so could not determine the intubation time according to the functional laryngeal view. [16] As C-L classification is not always sensitive or specific in predicting difficult intubation, Cook reported a new practical classification of the laryngeal view according to the degree of intubation difficulty. [16] However, our tube angle modification was successful in both C-L grades II and III. Third, our intubation time did not include the time needed to advance the tip of the double-lumen tube into the target bronchial position. However, once the stylet was removed from the double-lumen tube, the shape of the tube in the group N and M should be the same from this time point and therefore, the times required to advance the tube to the target bronchus between two groups also should be the same. Fourth, the usefulness of our method was restricted in patients with C-L grade I-III. Because only one patient showed C-L grade IV, it was impossible to evaluate the effectiveness of our method in patients with C-L grade IV in this study. However, considering that the alternative intubating devices are frequently used in patient with C-L grade IV instead of direct laryngoscopy, our method would be still useful in clinical practice. Fifth, we modified the tube angle during the mask ventilation after anesthesia induction. Although tube angle-modification did not take more than 15 seconds in our study, it would be more practical and convenient to modify the tube prior to anaesthesia induction so as to avoid any ventilation halting or additional need of support. Finally, despite our efforts, the present study was not strictly double-blinded due to the possibility that the performer was aware of the tube shape during tracheal intubation. However, it is impossible to achieve a perfect double-blinded design in this kind of study. [22] Thus, we considered the present study to be single-blinded.

In conclusion, in this randomized controlled trial, intubation with an individually angle-modified double-lumen tube reduced the time needed and the number of intubation attempts for successful tracheal intubation in patients with C-L grades I-III. The simple method with tube angle modification could be useful in double-lumen tube intubation in clinical practice.

## Supporting Information

S1 File CONSORT Checklist. (DOC)

S2 File Clinical Research protocol (Original language version). (DOC)

S3 File Clinical Research protocol (English language version). (DOC)

S4 File Data set of our study. (XLSX)
