# Supplementary material for: The Fast and Easy Way for Double-Lumen Tube Intubation: Individual Angle-Modification
Source: PLoS One. 2016 Aug 18;11(8):e0161434. doi: 10.1371/journal.pone.0161434 (PMC4990269; doi:10.1371/journal.pone.0161434)
Supplement: S2 File — (DOC) [file pone.0161434.s002.doc]

**임상시험 계획서**

**1. 임상시험의 명칭 및 단계**

이중관 기관내관 튜브 삽입 시 개별적으로 조절된 각도의 튜브와 통상적인 각도의 튜브의 비교: A randomized clinical trial

(Comparison of individually modified angled tube and conventional angled tube in double-lumen endotracheal tube intubation: A randomized clinical trial.)

**2. 임상시험 실시기관명 및 주소**

삼성서울병원 마취통증의학과

서울시 강남구 일원동 50 삼성서울병원 마취통증의학과

**3. 임상시험책임자, 담당자 및 공동연구자의 성명 및 직명**

임상시험책임자: 마취통증의학과 조교수 이종환

담당자 및 공동연구자: 마취통증의학과 임상강사 민정진

**4. 임상시험의 배경**

흉부 수술을 받는 환자에서 일측 폐환기를 위해 요구되는 이중관 기관내관 튜브의 삽입은 일반적으로 단일 내관 기관 튜브의 삽입 보다 난이도가 높은데, 이는 이중내관 기관 튜브의 외경이 더 굵고 단단하며, 특이적인 튜브 굴곡 등의 특성을 가지기 때문이다.

일반적으로 기관 삽관 시에는 환자의 oral-, pharyngeal-, laryngeal axis를 되도록 일직선이 되게 만들어 기관 삽관을 위한 이상적인 시야를 확보하기 위해 환자의 후두부를 10cm정도 높이고 머리를 신전시키는 Sniffing position을 시행한다. 하지만 환자마다 해부학적, 기능적 차이가 있기 때문에 모든 환자에서 oral axis와 pharyngolaryngeal axis가 항상 일치하지는 않는다.

특히나 이중 내관 기관 튜브는 단일내관 튜브에 비해 외경이 크고, 단단하기 때문에 기관 삽관 후 발생할 수 있는 hoarseness, sorethroat, dysphagia등의 합병증의 발생 위험도 더 높은데, 이중관 기관 튜브의 삽입 난이도를 높이는 튜브 인자들 중 튜브 외경이나 재질은 바꿀 수 없지만, 튜브 굴곡은 내부에 삽입되어 있는 stylet을 이용하여 조절할 수 있다. 이중관 기관 튜브의 굴곡을 환자마다의 해부학적, 기능적인 특성에 따라 개별적으로 조절한다면 기존에 제조사에서 제공하는 상태의 튜브에 비해 보다 신속하고, 안전한 기관 삽관이 가능할 것으로 생각된다.

**5. 연구 목적**

이중관 기관튜브의 삽관 환경과 발관 후 기관삽관과 관련된 부작용의 발생에 있어 환자에 따라 개별적으로 각도를 변형시킨 이중관 기관튜브와 별도의 조작을 가하지 않은 기존의 튜브 간의 차이를 비교해보고자 한다.

**6. 피험자의 선정기준, 제외기준, 목표한 피험자의 수 및 그 근거**

1) 선정 기준

예정된 흉부 수술을 받는 이중관 기관내관 튜브 삽입이 필요한 20세 이상 성인 환자중

2) 제외 기준

-경추 손상이나 움직임 제한, 류마티스 관절염 등 경추 질환을 가진 환자

-구강 및 후인두에 위치한 폐쇄성 종양이나 이물질을 가진 환자

-임산부

- Rapid sequence intubation이 요구되는 환자

-흔들리거나 약한 치아로 직접후두경 사용이 어려운 환자

3) Screening

선정 기준에 해당하면서 제외기준에 해당사항이 없는 환자

4) 중지/탈락 기준

피험자가 연구 중지/탈락을 요구하는 경우 어느 시점에서든 연구 중지/탈락 가능

**6. 목표한 피험자의 수**

이중관 기관내관 튜브의 삽입 성공에 걸리는 시간을 측정하여 두 군간 평균 10초 (표준편차 15) 이상 차이가 날 때 두 군간 유의한 차이가 있다고 가정하고, α값 0.05, power 90%로 계산했을 때, 한 군당 49명이 필요하다. 여기에 10%의 탈락율을 고려하면 각 군당 약 54명의 피험자가 필요하다.

- Modified angle군: 54명

- Conventional angle군: 54명

**7. 임상시험기간**

2014년 IRB 통과 직후 12개월

**8. 임상시험방법**

대상자 선정

전신마취 일측 폐환기가 요구되는 예정된 흉부 수술을 시행받는 환자들 중 상기된 선정기준에 부합되고 피험자가 동의할 경우 대상자로 선정한다.

피험자 동의확보

별첨된 설명문과 동의서에 따라 연구 담당자가 환자에게 설명한 후 서면 동의를 받는다.

무작위 배정

대조군: 이중관 기관 튜브 삽입 시 별도의 조작 없이 기존의 이중관 기관 튜브를 사용하는 군

시험군: 이중관 기관 튜브 삽입 시 개별적으로 각도가 조절된 이중관 기관 튜브를 사용하는 군

피험자 등록 전 환자의 진료에 관여하지 않은 의사가 대조군(A)과 시험군(B)을 각각 2명씩 포함하는 크기가 4인 블록(AABB, BBAA, ABAB, BABA, ABBA, BAAB) 20개로 구성된 블록 무작위 배정표를 난수표에 따라 미리 작성하고, 이 배정표에 따른 순서대로 피험자를 대조군이나 시험군에 배정하여 시험을 진행한다. 무작위 배정 비율은 시험군과 대조군에 1:1로 배정되도록 한다. 단, 무작위 배정표는 피험자가 시험에 등록되기 전에 연구에 참여하는 피험자를 볼 수 없는 연구와 독립된 제 3자에 의해 만들도록 하며 운영, 관리되어 Allocation concealment를 유지하도록 한다.

임상시험의 시행 계획

마취 전 평가를 위한 환자 방문 시 mouth opening, modified Mallampati score, and thyromental distance를 기록한다. 수술장에서 기관 삽관 시 이용하는 이중관 기관 튜브의 각도를 제외한 모든 임상 처치는 양 군에서 동일하게 시행한다. 통상적인 마취방법을 이용하여 의식 소실과 근육 이완을 유도 후 마스크 용수 환기 동안, Modified angle 군에서는 환자를 sniffing position으로 만든 후 지정된 시술자에 의해 tube angle modification이 이루어진다. 이중관 끝을 환자의 cricoid cartilage 상단에 놓고, 접이 자를 이용하여 i) oral axis와 pharyngolaryngeal axis가 이루는 각도에 따라 이중관 기관 튜브의 굴곡을 변형시킨다.


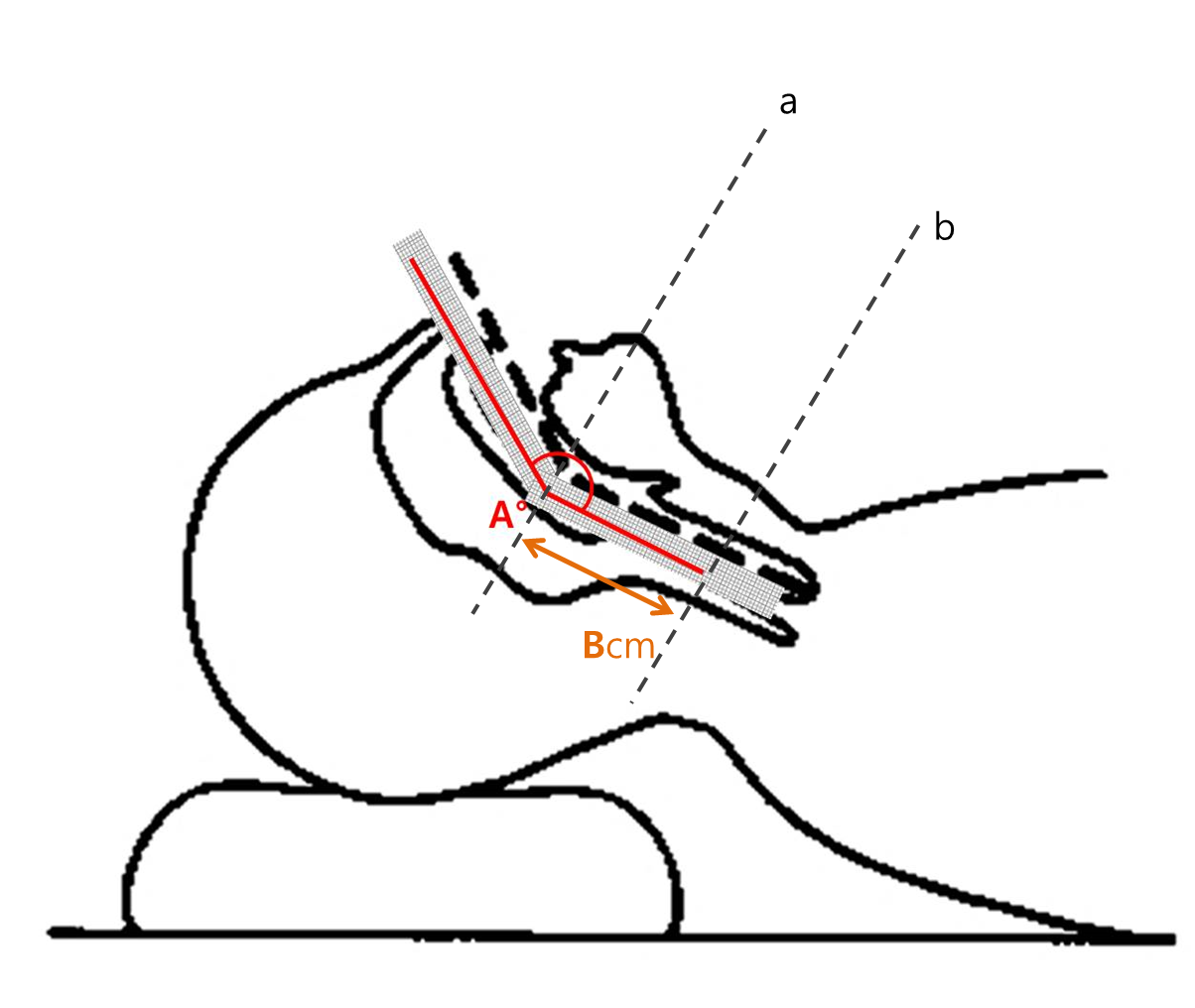


**Figure 1.**

a: joint point of the two axis

b: cricoid cartilage level

A: Angle between oral axis and the pharyngolaryngeal axis

B: Length between a to b

충분한 근이완 후 Macintosh Laryngoscopy를 이용하여 대조군에서는 별도의 조작을 가하지 않는 튜브를, 시험군에서는 위의 측정값대로 튜브 끝에서부터 Bcm 되는 지점에서 A의 각도로 굴곡을 변형시킨 이중내관 기관 튜브를 삽입한다. 이때 기관 내 삽관은 튜브각 변경을 알지 못하는 정해진 시술자 (민정진, 이종환)에 의해서만 시행되도록 하며, 독립된 기록자가 삽입 성공에 걸린 시간과 삽입 횟수, 혈액학적 수치, 들어간 심혈관 약제 등을 기록한다. 삽입 시작 시간은 Laryngoscopy를 입 안으로 거치시키기 시작한 시점, 삽입 끝 시간은 이중관 기관 튜브가 성문을 통과하는 때로 한다. 성문 통과 후에는 양 군 모두에서 동일하게 튜브를 좌측으로 90도 회전시키고 기관지 내시경을 이용하여 위치를 확인한다. 첫 시도에 삽관 실패 시, 추가 시도 횟수는 2회까지 허용한다. 튜브 위치 확인 및 고정 후, 구강 및 후인두 손상이나 출혈이 없는지 관찰하고 기록한다.


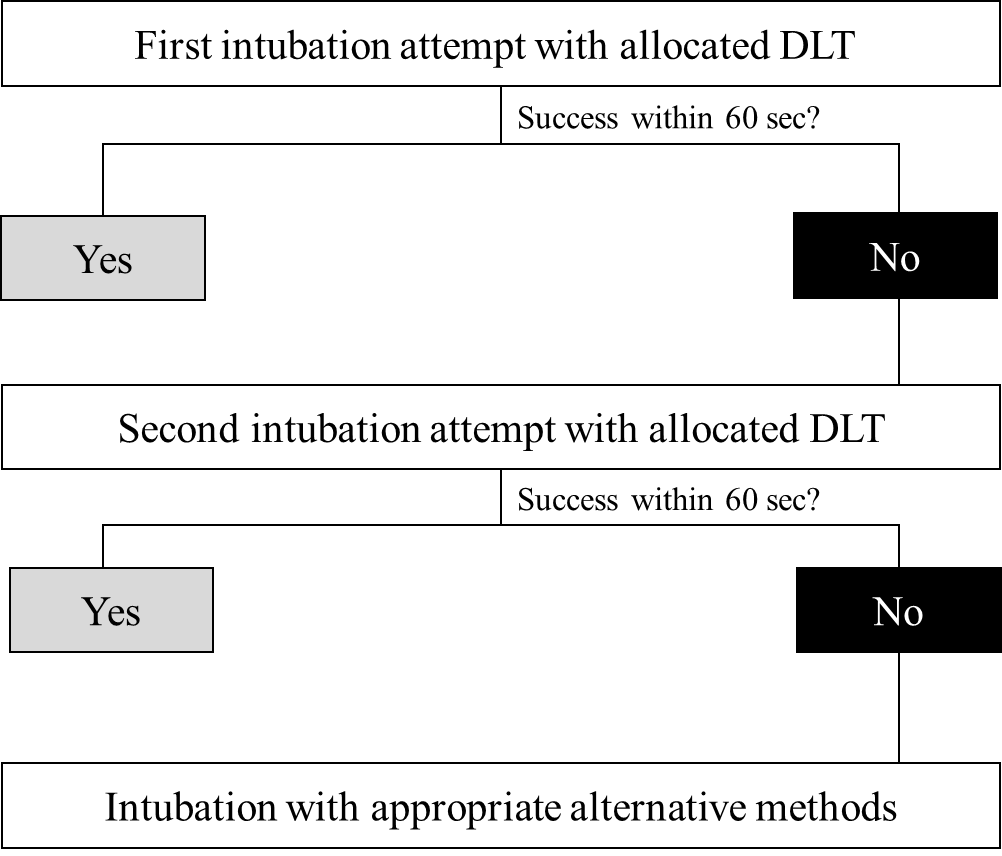


수술이 종료되고 회복실 입실 30분 후와 수술 종료 24시간이 지난 시점에 군 배정을 알지 못하는 독립적인 관찰자에 의하여 인후통, 쉰목소리 유무, 있다면 심한 정도 (none-mile-moderate-severe)를 기록하도록 한다.

**9. 관찰항목/임상검사항목 및 관찰검사방법**

환자 정보

나이, 성별, 키, 체중, 체질량 지수 (Body mass index), ASA class, Mallampatti grade, Neck extension가능 정도, 총 마취 시간, 수술 종류, 삽입한 이중관 기관 튜브 사이즈와 굴곡 변형 시 측정된 A, B값

기관 삽관 시 DATA

삽관 성공까지 걸린 시간, external laryngeal manipulation 필요 여부, 삽관 시도 횟수, 기관 삽관 시 hemodynamic data (혈압, 맥박), 기관 삽관 시기에 사용된 심혈관계 작용 약물의 종류와 양, Cormack-Lehane grade, Intubation Difficulty scale, any oropharyngeal trauma

수술 후 증상 (수술 후 30분 후, 24시간 경과 후)

Sore throat, hoarseness발생 유무와 중증도

**10. 효과 평가기준, 평가방법 및 해석방법 (통계분석방법)**

1) Primary endpoint: 기관 삽관 시작으로부터 삽관 성공까지 걸린 시간에 있어 양 군에서 유의한 차이가 있는지 관찰한다.

2) Secondary endpoint: 수술 후 발생한 상기도 후유증의 발생의 빈도에 양 군에서 유의한

3) 통계분석 방법

통계 프로그램은 SPSS 20.0을 사용한다. 각 항목에 대해 양 군 간 비교분석을 아래와 같은 방법으로 시행한다. 자료의 정규분포를 확인 후, 기관삽관 시작부터 성공 시점까지 걸린 시간은 Student's *t*-test 또는 Mann-Whitney U test를 이용하여 비교 분석한다. 성별, 수술명, 수술 종료 후 인후통, 쉰목소리 발생 여부와 심한 정도는 Chi-square test 또는 Fisher's exact test를 이용하여 비교 분석한다. C-L classification과 삽관 휫수의 연관성은 Pearson’ correlation을 이용하여 분석한다. P 값이 <0.05인 경우 통계학적으로 유의한 것으로 판단한다.

**증례기록서 양식 및 동의서 양식은 아래 별첨**

**11. 피험자의 안전보호에 관한 대책**

본 시험에 참여한 모든 환자를 대상으로 안전성 평가를 실시한다.

연구 시행 중에 이상반응이 발생했을 경우, 연구에 사용된 시험 방법과의 인과 관계 유무와 모든 이상 반응을 기록하고 추후 중증도, 중대성, 기간, 그리고 시험 방법과의 인과관계를 평가한다. 이상반응에 대한 처치 및 결과 역시 기록한다. 이상 반응은 시험 기간 중의 계획된 검진과 검사의 소견 이외에도 비정상적인 검진이나 필요에 따른 추가적인 검사와 검진에 의해 평가하고 즉각적인 조치를 시행한다. 임상병리검사 자료에 대해서는 변수의 특성에 따라 치료 전, 후의 군내 비교 등 적절한 통계적 방법을 이용하여 분석하고, 이상반응의 빈도, 발현율, 각각의 목록, 심각한 정도 및 시험 방법과의 인과관계 등을 제시하며, 필요한 경우 그래프 형태로 보고한다.

1)중증도

이상반응은 아래의 정의에 따라 경증, 중등증, 중증으로 구분된다.

경증은 일반적이고 일시적이고 일상적인 활동을 방해하지 않는다.

중등증은 약간의 불편함을 초래하거나 일상적인 활동을 방해한다.

중증은 일상적인 활동을 수행할 수 없다.

2)인과 관계

인과 관계는 ‘관련 없을 것으로 생각됨’, ‘관련 있을 가능성 있음’, ’가능성 많음’, ’명백히 관련 있음’, 또는 ‘관련성을 확인하기 어려움’으로 구분된다.

마취 유도 및 유지 과정은 숙련된 마취과 전문의가 함께 감시하므로 합병증이 발생하더라도 적절한 조치를 취하게 되므로 환자에게 추가 위험의 가능성은 매우 적을 것이라 생각한다.

피험자의 의무기록을 포함한 사적정보의 보호를 위해 다음의 내용을 실천한다.

1. 데이터는 미리 준비한 case report form에 기록하며, 즉시 잠금장치가 있는 연구실에 비밀번호가 걸린 파일로 보관한다.
2. 연구파일에 접근할 수 있는 사람은 권한을 가진 일부 연구원으로 제한한다. (연구원의 소속: 마취통증의학과, 성명: 이종환, 민정진)
3. 수집되는 자료의 불필요한 개인식별자는 제거한다. 특히, 증례기록서에는 환자의 이름, 주민등록번호, Chart No. 등을 기재하지 않도록 하며, 신상정보와 연결된 식별자 코드는 별도로 관리한다.

본 연구는 병원 윤리위원회의 윤리규정을 준수할 것이다.

**References**

1. Campos JH. Which device should be considered the best for lung isolation: double-lumen endotracheal tube versus bronchial blockers. Curr Opin Anaesthesiol 2007;20:27-31.

2. David Michael Stout et al. Correlation of endotracheal tube size with sore throat and hoarseness following general anesthesia. Anesthesiology 1987;67:419-21.

3. The Intubation Difficulty Scale. Anesthesiology 1997;87-1290-7.

4. Zhong T. et al. Sore throat or hoarse voice with bronchial blockers or double-lumen tubes for lung isolation: a randomised, prospective trial. Anaesth Intensive Care 2009;37:441-6.

5. Knoll H et al. Airway injuries after one-lung ventilation: a comparison between double-lumen tube and endobronchial blocker: a randomized, prospective, controlled trial. Anesthesiology 2006;105:471-7

6. McHardy FE et al. Postoperative sore throat: cause, prevention and treatment. Anaesthesia 1999;54:444-53.

7. Woo P. et al.  Diagnostic-value of stroboscopic examination in hoarse patients. Journal of Voice 1991;5:231-8.

8. Capan LM et al.  Succinylcholine-induced postoperative sore throat. Anesthesiology 1983;59:202-6.

9. Simon Wasem et al. Comparison of the Airtraq and the Macintosh laryngoscope for double-lumen tube intubation. EJA 2013; 30:180-186.

10. T. Russell et al. A randomised controlled trial comparing the GlideScope and the Macintosh laryngoscope for double-lumen endobronchial intubation. Anaesthesia 2013; 68:1253-1258.

[별첨 1]

**Case Report Form Case No.**

**The fast and easy way for double-lumen tube intubation: Individual angle-modification:**

**A randomized clinical trial**

Study date: Date of randomization:

Log No.:

Study group: Modified angle ( ) / Conventional angle ( )

Op name:

Comments:

| Age | | | |  | | | | | | | | | Sex | | | | | | | | | M / F | | |
| --- | --- | --- | --- | --- | --- | --- | --- | --- | --- | --- | --- | --- | --- | --- | --- | --- | --- | --- | --- | --- | --- | --- | --- | --- |
| ASA class | | | |  | | | | | | | | | Ht/ Wt/ BMI | | | | | | | | |  | | |
| **Airway characteristics** | | | | | | | | | | | | | | | | | | | | | | | | |
| Inter-incisor distance | | | | | | cm | | | | | Passive mouth open | | | | | | | | | | | | cm | |
| Mallampati grade | | | | | | I / II / III / IV | | | | | Thyromental distance | | | | | | | | | | | | cm | |
| Cormack Lehane | | | | | | I / II / III / IV | | | | | Cormack Lehane  (with BURP) | | | | | | | | | | | | I / II / III / IV | |
| **Hemodynamic data during intubation** | | | | | | | | | | | | | | | | | | | | | | | | |
| 1^st^ Attempt | | | | | | | | | | | | | | | | | | | | | | | | |
|  | | | baseline | | | | | 1min | | | | | | | 2min | | | | 3min | | | | | 5min |
| HR | | |  | | | | |  | | | | | | |  | | | |  | | | | |  |
| Systolic BP | | |  | | | | |  | | | | | | |  | | | |  | | | | |  |
| Any drugs | | |  | | | | |  | | | | | | |  | | | |  | | | | |  |
| 2^nd^ Attempt | | | | | | | | | | | | | | | | | | | | | | | | |
|  | | | baseline | | | | | 1min | | | | | | | 2min | | | | 3min | | | | | 5min |
| HR | | |  | | | | |  | | | | | | |  | | | |  | | | | |  |
| Systolic BP | | |  | | | | |  | | | | | | |  | | | |  | | | | |  |
| Any drugs | | |  | | | | |  | | | | | | |  | | | |  | | | | |  |
| 3^rd^ Attempt | | | | | | | | | | | | | | | | | | | | | | | | |
|  | | | baseline | | | | | 1min | | | | | | | 2min | | | | 3min | | | | | 5min |
| HR | | |  | | | | |  | | | | | | |  | | | |  | | | | |  |
| Systolic BP | | |  | | | | |  | | | | | | |  | | | |  | | | | |  |
| Any drugs | | |  | | | | |  | | | | | | |  | | | |  | | | | |  |
| **Intubation data** | | | | | | | | | | | | | | | | | | | | | | | | |
| Tube size | | | | | 32 / 35 / 37 / 39 / 41 | | | | | | | | | | | | | | | | | | | |
| 1st Attempt | | | | | | | | | | | | | | | | | | | | | | | | |
| Time of start | | | | |  | | | | | | | | | Time of end | | | | | |  | | | | |
| Intubation success | | | | | Success / Fail | | | | | | | | | BURP | | | | | | Y / N | | | | |
| CL grade | | | | | I / II / III / IV | | | | | | | | | | | With BURP: I / II / III / IV | | | | | | | | |
| Any oropharyngeal trauma | | | | | | | | | | Y / N ( ) | | | | | | | | | | | | | | |
| Blood at laryngoscope blade | | | | | | | | | | Y / N ( ) | | | | | | | | | | | | | | |
| 2nd Attempt | | | | | | | | | | | | | | | | | | | | | | | | |
| Time of start | | | | |  | | | | | | | | | Time of end | | | | | |  | | | | |
| Intubation success | | | | | Success / Fail | | | | | | | | | BURP | | | | | | Y / N | | | | |
| CL grade | | | | | I / II / III / IV | | | | | | | | | | | With BURP: I / II / III / IV | | | | | | | | |
| Any oropharyngeal trauma | | | | | | | | | | Y / N ( ) | | | | | | | | | | | | | | |
| Blood at laryngoscope blade | | | | | | | | | | Y / N ( ) | | | | | | | | | | | | | | |
| 3rd Attempt | | | | | | | | | | | | | | | | | | | | | | | | |
| Time of start | | | | |  | | | | | | | | | Time of end | | | | | |  | | | | |
| Intubation success | | | | | Success / Fail | | | | | | | | | BURP | | | | | | Y / N | | | | |
| CL grade | | | | | I / II / III / IV | | | | | | | | | | | With BURP: I / II / III / IV | | | | | | | | |
| Any oropharyngeal trauma | | | | | | | | | | Y / N ( ) | | | | | | | | | | | | | | |
| Blood at laryngoscope blade | | | | | | | | | | Y / N ( ) | | | | | | | | | | | | | | |
| **Postoperative data** | | | | | | | | | | | | | | | | | | | | | | | | |
|  | | | | | | | Postoperative 30 min | | | | | | | | | | Postoperative 24 hr | | | | | | | |
| Sore throat | | | | | | |  | | | | | | | | | |  | | | | | | | |
| Hoarseness | | | | | | |  | | | | | | | | | |  | | | | | | | |
| 0 : none, 1: mild, 2: moderate, 3: severe | | | | | | | | | | | | | | | | | | | | | | | | |
|  | **임상 시험 책임자 서명** | | | | | | | | | | | | | | | | | | | | | | | |
|  | 본인은 위의 피험자로부터 적절한 방법에 따라 문서 동의를 얻었으며, 본 증례기록지에 기록된 모든 내용이 위의 피험자로부터 정확히 얻어진 결과임을 확인합니다.  본인 또는 본인이 위임하는 자는,   1. 본 증례 기록지의 내용을 모두 검토하였으며, 2. 그 내용이 정확하고 3. 기록된 날짜에 시행된 검사 또는 측정의 결과임을 확인합니다. | | | | | | | | | | | | | | | | | | | | | | | |
|  | 시험자 성명 |  | | | | | | | 서명 | | |  | | | | | | 날짜 | | | 년 월 일 | | | |

**연구과제 피험자 동의서**

(삼성서울병원)

| 연구 제목 | 이중관 기관내관 튜브 삽입 시 개별적으로 조절된 각도의 튜브와 통상적인 각도의 튜브의 비교: A randomized clinical trial |
| --- | --- |
| 연구책임자 | 삼성서울병원 마취통증의학과 조교수 이종환 02-3410-1928 |
| 연구담당자 | 삼성서울병원 마취통증의학과 임상강사 민정진010-8666-1775 |

*** 만일 본 연구에 문의사항이 있으시거나, 위험이나 불편 또는 손상이 발생할 경우, 상기 연구자에게 연락하여 주시기 바랍니다.**

1. **참여 권유**

본 시험책임자는 귀하로부터 임상시험 참여에 대한 동의를 받고 이를 문서화 할 때 관련규정을 준수하며 헬싱키선언에 근거한 윤리적 원칙을 바탕으로 합법적인 절차를 따를 것입니다. 귀하는 본 임상시험에 참여할 것인지 여부를 결정하기 전에, 이 동의서를 신중하게 읽어보셔야 합니다. 연구의 참여는 본인의 자발적인 의사에 의해서만 가능합니다. 만일 참여를 원하지 않으신다고 하여도 추후의 치료과정에는 어떤 영향도 미치지 않을 것입니다. 이 시험에 대하여 설명한 이 문서를 읽으면서 어떤 질문이라도 할 수 있습니다. 충분한 시간을 가지고 결정한 후 서명해 주십시오. 귀하와 이 문서에 대해서 설명한 시험책임자(또는 시험책임자의 위임을 받은 자)가 본 서식에 서명하고 자필로 해당 날짜를 기재해야 합니다. 귀하의 서명은 귀하가 본 시험에 대해 그리고, 위험성에 대해 설명을 들었음을 의미합니다. 또한, 이 문서에 대한 귀하의 서명은 귀하께서 자신(또는 법정대리인)이 본 시험 참가를 원한다는 것을 의미합니다.

1. **본 임상시험은 연구목적으로 수행됩니다**

폐수술을 위해서는 수술 시야 확보를 위해 일측 폐환기가 필요한데, 이를 위해서는 양측 폐의 환기를 각각 조절할 수 있는 이중관 기관 튜브의 삽입이 필요합니다. 이중관 기관 튜브는 외경이 더 굵고 단단하여 삽관 시 난이도가 단일관 튜브에 비하여 높은 편입니다. 그런데 환자분에 따라 해부학적, 기능적인 차이가 있을 수 있기 때문에 본 연구에서는 기관 삽관에 적합한 포지션을 취한 뒤, 입-후두-기관으로 이어지는 굴곡을 환자분에 따라 개별적으로 변화시킨 튜브와 기존에 제조사에서 제공하는 그대로의 튜브를 비교하여 기관 삽관 환경 및 발관 후 합병증의 발생에 차이가 있는지를 분석하고자 하는 연구입니다.

**3. 연구 방법 및 예측 효능, 효과**

귀하께서 연구에 적합한 대상으로 확인되고 참여에 동의하셨다면 본 시험에 참여하게 됩니다. 귀하가 본 연구에 참여함으로 인해 현재 통상적으로 행하여지는 수술과 마취방법에서 연구에 사용되는 튜브의 굴곡 이외에 연구에 참여하지 않은 시험 대상자들과의 차이점은 없습니다.

연구에 참여하는 모든 대상자 분은 귀하의 해부학적, 기능적 차이에 따라 튜브의 굴곡을 변형시키는 군과 기존의 튜브를 그대로 사용하는 군으로 무작위 배정됩니다. 무작위 배정이란 두가지 시험군 중에 시험대상자 분이 어떤 군으로 배정이 될 지가 연구자의 의지가 개입되지 않도록 우연에 의해 결정되는 방법이며, 참여하시는 분들이 어느 편으로도 치우치지 않고 공정하고 중립적인 대우를 받게 하고 연구 결과의 내적 타당도를 높이기 위함입니다. 구체적인 방법으로는, 대조군(A)과 시험군(B)을 각각 2명씩 포함하는 크기가 4인 블록(AABB, BBAA, ABAB, BABA, ABBA, BAAB) 20개로 구성된 블록 무작위 배정표를 컴퓨터 프로그램을 이용하여 미리 작성하고, 이 배정표에 따른 순서대로 피험자를 A또는 B군으로 배정하게 됩니다. 기관 삽관은 실험에 참여하는 모든 대상자에서 군에 상관없이 숙련된 마취과 전문의에 의해 시행됩니다. 귀하의 개별적인 해부학적 및 기능적인 특성을 고려하여 이중관 기관 튜브의 굴곡을 변형시키면 보다 신속하고 안전하게 삽관을 시행할 수 있을 것으로 기대합니다.

2013년 4 **4. 임상시험 참여로 인하여 예견되는 부작용이나 불편사항**

튜브각을 조절하는 군에서, 삽관 과정 중 발생할만한 부작용은 일반적으로 후두경을 이용한 기관 삽관 시에 발생할 수 있는 부작용으로 동일할 것으로 생각됩니다. 후두경을 이용한 기관 삽관 시에 발생 가능한 부작용으로는 구강 및 후인두 부위의 연부 조직의 손상, 치아 손상, 후인두 벽 자극 시에 미주신경 자극으로 인한 서맥 등이 있습니다. 다만, 튜브가 성문을 통과한 후 stylet을 제거할 때, 튜브 각도를 변경한 군에서 각도가 90도에 가까울 경우 보통의 각도에서 보다 제거하는데 보다 물리적 힘과 시간이 걸릴 수 있으나, 삽관 난이도가 어려운 환자일수록 변경 각도가 직각에 가까울 것이므로 삽관 시에 예상되는 시간 소요나 연부조직 손상 등에 비하면 환자에게 미치는 합병증은 미미할 것으로 예상됩니다. 또한 추가적인 부작용의 발생 가능성이 극히 적지만 발생할 수 있기 때문에 이런 경우 숙련된 마취과 전문의가 적절한 조치를 취하게 될 것입니다.

**5. 본 임상시험에 참여함으로써 기대되는 이익**

앞서 설명하였듯이 본 연구참여로 인해 금전적으로나 다른 어떠한 형태로도 귀하가 이익이나 손해는 없습니다. 본 연구의 결과가 나왔을 때, 이를 학문적으로 학계에 보고하여 향후 진료와 환자 안전에 도움이 될 수 있을 것입니다.

**6. 예상 참여기간 및 본 시험에 참여하는 대략의 전체 피험자 수**

정규로 예정된 일측 폐환기가 요구되는 흉부 수술을 받는 20세 이상 성인을 대상으로 108여명의 대상자 분들께서 본 연구에 참여할 예정이며 동일한 비율로 각 군에 배정받게 됩니다.

**7. 임상시험과 관련된 손상이 발생하였을 경우의 보상/배상이나 치료방법**

본 임상시험과 관련하여 만약 손상이 발생하여 응급조치가 필요할 경우, 삼성서울병원에서 행해지는 처음 24시간 동안의 응급조치에 필요한 치료비에 대해서 삼성서울병원에서 부담합니다.

**8. 임상시험에 참여함으로써 받게 되는 금전적 보상의 여부 및 참여 정도에 따른 조정 정도 또는 임상시험에 참여함으로써 추가적으로 발생이 예상되는 비용**

본 임상시험에 참여함으로써 제공되는 금전적인 보상은 없으며 임상시험 참여로 추가로 발생이 예상되는 연구와 연관된 비용은 없습니다.

**9. 임상시험 지속 참여 의지에 영향을 줄 수 있는 새로운 정보**

이 연구에 대한 참여는 전적으로 귀하의 선택입니다. 이 연구에 지속적으로 참여할 귀하의 의지에 영향을 줄 수 있는 새로운 정보가 수집되면 적시에 귀하 또는 대리인에게 알려드릴 것이나 본 연구는 일정 시점의 관찰로서 지속 참여를 요구하지 않습니다.

**10. 자유의사에 의한 시험 참여 동의 및 철회 및 연구중단 이후의 절차**

본 임상시험에의 참여는 전적으로 자발적입니다. 임상시험에 참가 여부를 결정하는 것은 귀하에게 달려있습니다. 만약 귀하가 임상 시험에의 참여를 결정하였더라도 귀하는 이유를 대지 않고 언제든지 임상시험을 자유롭게 중단할 수 있습니다. 만약 귀하가 임상시험에의 불참 또는 참여 중단을 선택하였더라도 이것이 향후 귀하의 의학적 치료에 어떠한 방식으로도 영향을 미치지 않을 것입니다. 만약 귀하가 임상시험에의 참여에 대한 동의를 철회하고자 결정하였다면 귀하의 시험 담당 의사는 최종 평가를 시행하고 보고서 양식에 데이터를 수집하는 것에 대한 귀하의 동의를 구할 것입니다. 그러나 귀하가 동의하지 않으면 귀하에 대한 어떠한 새로운 자료도 수집하지 않을 것이며 연구대상자의 개인정보가 담긴 모든 데이터 또한 요청 시 폐기될 것 입니다.

**11. 신분의 비밀 보장**

귀하의 신원을 파악할 수 있는 기록은 기밀유지가 되고 공개적으로 열람되지는 않습니다. 다만, 관련법이나 규정에 의해 허용되는 범위 안에서 임상시험의 실시절차와 자료의 신뢰성을 검증하기 위해 모니터요원, 점검을 실시하는 자, 심사위원회 및 정부기관에 의해 귀하의 의무기록이나 자료를 직접 열람할 수 있지만 이 경우에도 최대한 기밀유지가 되도록 할 것입니다. 귀하께서 본 동의서에 서명함은 이러한 자료의 직접 열람을 허용한다는 것을 의미하며, 임상시험의 결과가 출판될 경우 귀하의 신원은 비밀 상태로 유지될 것입니다.

**12. 피험자로서의 권익에 관한 정보 제공**

본 임상시험은 본원 피험자의 권리, 안전, 복지를 보호할 책임이 있는 기관윤리심의위원회(IRB)에 의해 승인되었으며 본 연구의 참가자(피험자)로서 귀하의 권리에 대해 질문이 있으시면 당원에서 지정한 “피험자보호 연구윤리 담당자(TEL 02-3410-2980)”에게 문의하실 수 있습니다. 본 시험에 참가하기로 선택하였다면 귀하는 서명된 동의서의 사본을 받게 될 것입니다.

**13. 개인 정보 보호에 관한 정보**

본 임상 연구의 참여로 인해 귀하의 성명, 성별, 연령과 같은 개인정보가 수집되어 지지만 해당 정보는 연구에 직접 이용되거나 필요로 하는 정보가 아니며 임상연구로 인해 획득된 귀하의 임상 정보에 연결하기 위한 목적으로만 사용됩니다. 수집된 정보는 1년간 사용되게 되며, 개인정보보호법에 따라 적절히 관리됩니다. 피험자는 개인정보의 수집, 이용 목적, 보유 및 사용기간, 수집하려는 개인정보 항목이 무엇인지 알 권리가 있으며, 개인 정보 수집을 거부할 권리가 있습니다.

본인은 본 동의서의 내용에 대해 설명을 들었고, 동의서 내용을 읽고 이해하였으며 본인이 궁금해하는 모든 질문에 대한 답변을 들었습니다.

본인은 자발적으로 본 연구에 참여하는 것에 동의하므로 동의서에 서명하며 동의 후에 **동의서 사**

**본을 제공받을 것임을 알고 있습니다.**

시험에 참여하는 피험자 성명: 서명 혹은 인

년 월 일

법정대리인 환자와의 관계 성명: 서명 혹은 인

년 월 일

설명한 의사 성명: 서명 혹은 인

년 월 일

본 동의서는 기관윤리심의위원회(IRB)에서 심의하여 사용을 승인한 동의서로.

SMC철인이 된 경우에만 유효합니다
