# Supplementary material for: The Fast and Easy Way for Double-Lumen Tube Intubation: Individual Angle-Modification
Source: PLoS One. 2016 Aug 18;11(8):e0161434. doi: 10.1371/journal.pone.0161434 (PMC4990269; doi:10.1371/journal.pone.0161434)
Supplement: S3 File — (DOC) [file pone.0161434.s003.doc]

**Clinical Research Protocols**

**1. Title**

Comparison of individually modified angled tube and conventional angled tube in double-lumen endotracheal tube intubation: A randomized clinical trial.

**2. Institution**

Department of Anesthesiology and Pain Medicine of Samsung Medical Center, Sungkyunkwan University School of Medicine

**3. Principal investigator and sub-investigators**

- Principal investigator: Dr. Jong-Hwan Lee, Assistant professor

- Sub-investigator: Jeong Jin Min, Se Hee Kang, Eunhee Kim, Sangmin M. Lee, Jong Ho Cho, Hong Kwan Kim

**4. Background**

A double-lumen tube is more difficult to insert than a single-lumen, although it has been generally accepted as a standard technique for lung isolation during thoracic surgery. Moreover, various videolaryngoscopic devices, in spite of the successful achievement of better laryngeal views, have failed to show the superiority to the direct laryngoscopy for the faster placement of double-lumen tube.

Basically, tracheal intubation is composed of three sequential steps: 1) the achievement of laryngeal view, 2) the delivery of tube to the glottis, and 3) the advancement of tube into trachea. Therefore, the ability to visualize the larynx might not be sufficient for the fast and successful intubation of a double-lumen tube. Moreover, considering the distinguishing characteristics of double-lumen tube, accurate delivery of the tube to the glottis might also be a crucial step, although a good laryngeal view is a common important step for tracheal intubation. Theoretically, modification of the tube shape according to each patient’s upper airway axes might facilitate tube delivery to the glottis and make inserting a double-lumen tube easier and faster.

**5. Aim**

We hypothesized that the individually angle-modified double-lumen tube is superior to the manufacturer-provided double-lumen tube with respect to the time and the number of attempts needed for successful intubation. The aim of this study was to evaluate the usefulness of individual angle-modification in patients requiring double-lumen tube intubation.

**5. Inclusion and exclusion criteria**

1) Inclusion criteria: Adult patients (≥ 20yrs) who required double-lumen tube insertion for elective thoracic surgery

2) Exclusion criteria: cervical spine disease that restricted head extension, such as rheumatoid arthritis with atlantoaxial subluxation or cervical disc disease, oropharyngeal obstructive disease; patients requiring rapid sequence intubation; presence of loose or vulnerable tooth; and pregnancy

3) Screening: Adult patients (≥ 20yrs) who required double-lumen tube insertion for elective thoracic surgery without exclusion criteria

4) Withdrawal: Patients who wish to withdraw from the study can withdraw at any time.

**6. Sample size calculation**

The primary outcome is intubation time. Assuming a meaningful difference of 10 sec in mean intubation time between the two groups with a standard deviation (SD) of 15 sec, sample size was determined with a power of 0.9 and a type 1 error of 0.05. Power analysis suggested that a minimum of 49 patients per group was required. Considering a dropout rate of 10 %, we enrolled 54 patients per group.

**7. Study period**

Twelve months from the approval of the IRB

**8. Study design & Methods**

Randomization

Patients are randomly allocated by an independent anesthesiologist into either a non-modified double-lumen tube group or an individually angle-modified double-lumen tube group according to the tube angle using a computer-generated random number table.

Preoperative airway assessment

During the preoperative visit, the airway will be assessed by examining mouth opening, modified Mallampati score, and thyromental distance. Mouth opening is defined as the distance between the upper and lower incisors or gingiva in edentulous patients and was measured in centimeters with the mouth fully opened. Mallampati classification is determined with the patient in a sitting position with the mouth fully open and tongue protruding without phonation. Thyromental distance is measured along a straight line from the thyroid notch to the lower border of the mandibular mentum with the head fully extended.

Tube angle modification

In group M (Tube angle modification): the tube will be modified in the sniffing position by anesthesiologist who assigned the groups as following steps. First, each airway axes will be estimated on the lateral side of an patient as follows: oral axis is estimated as a straight line drawn from the tip of the upper incisors parallel to the hard palate; laryngeal axis is estimated as a straight line from the cricoid cartilage near-parallel to a patient’s anterior neck line; pharyngeal axis is estimated as a straight line drawn from the upper margin of cricoid cartilage with an angle of 10 - 15º posteriorly to the laryngeal axis; Second, the distal tip of tube is placed at the upper margin of the cricoid cartilage. Third, in order to create a fluent curve, the tube is bent at the intersection between the estimated oral and pharyngeal axes while maintaining the alignment of upper and lower parts of tube with the estimated oral and pharyngeal axes, respectively. All three steps for the tube modification were implemented during the mask ventilation and took no more than 15 seconds.

Anesthesia and Intubation

Anesthetic drugs and techniques will be standardized in all patients. Each patient is laid on the operating table with a 7-cm-high cushion under the head. Non-invasive blood pressure, three-lead ECG, and oxygen saturation are monitored. Anesthesia is induced with intravenous thiopental sodium (5 mg/kg) and continuous infusion of remifentanil (0.2-0.3 mcg/kg/min). Neuromuscular blockade is obtained with intravenous rocuronium bromide (0.6 mg/kg). Lungs are ventilated via a face mask with 5 vol% sevoflurane. Sufficient neuromuscular blockade is assessed by Train-of-Four monitoring. In the sniffing position, patients are intubated with a double-lumen tube according to group using a Macintoch laryngoscope blade. Once the stylet in the bronchial lumen was removed when the tip of the tube was past the glottis, the tube is rotated 90° counterclockwise.

One of 2 experienced anesthesiologists not aware of the exact procedure, remained outside the operating room until the tube modification is completed and then performed all intubations. Intubation data including intubation attempts, application of the BURP (backward, upward, and rightward pressure on the larynx) maneuver, and any oropharyngeal injuries will be recorded by an independent anesthesiologist. The best laryngeal view obtained with or without BURP is recorded by an anesthesiologist performing the intubation according to the Cormack and Lehane (C-L) grading scale. The intubation difficulty scale score was also calculated.

Intubation time and protocol


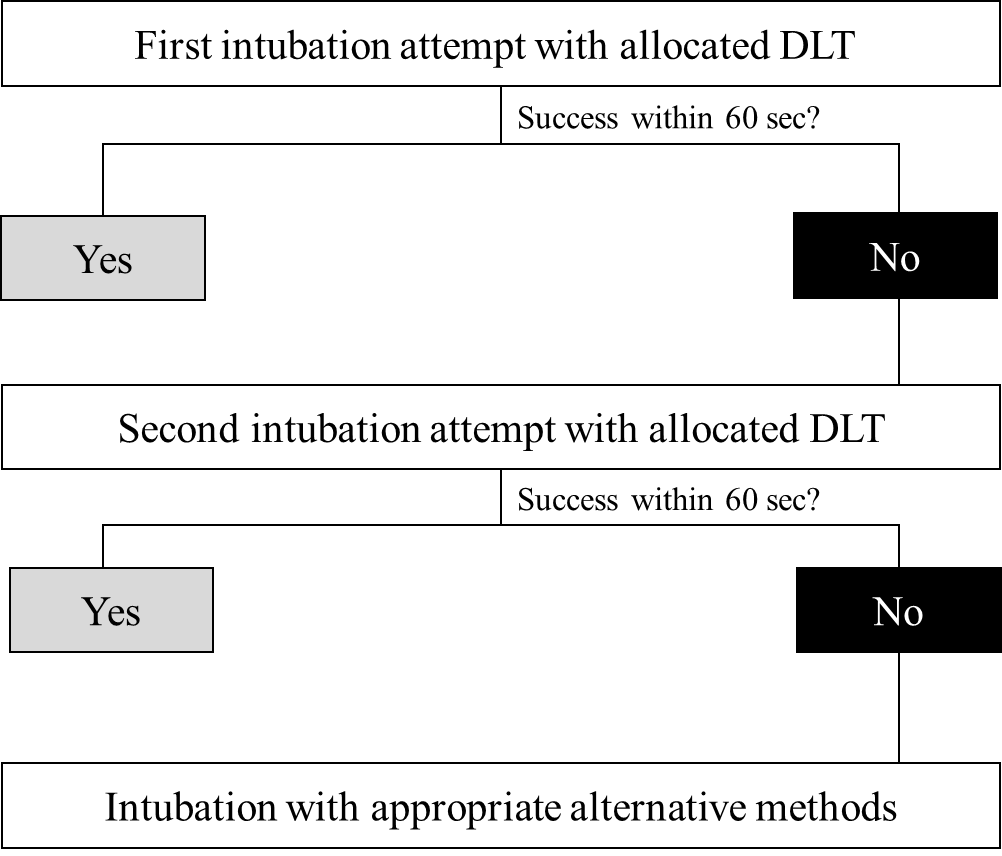


**9. Variables**

Patient characteristics

Age, sex, height, weight, Body Mass Index, ASA class, Mallampatti grade, Neck extension, Inter-incisor distance, passive mouth opening, Thyromental distance

Intubation data

Used tube size, Number of attempts, Time to intubation, need for external laryngeal manipulation, hemodynamic data in peri-intubation period, Cormack-Lehane grade, Intubation Difficulty scale, any oropharyngeal trauma, Blood at laryngoscopy blade, any other technique used for intubation

Postoperative complications (Postoperative 30 min and 24h) by H. K. Kim or J. H. Cho

Severity of Sore throat and hoarseness (0:none, 1:mild, 2: moderate, 3: severe)

**10. End points and Statistics**

The primary outcome is intubation time.

After normality check for data distribution with Kolmogorov-Smirnov tests, Student’s *t*-test or Mann-Whitney *U*-test will be used to compare the continuous variables between the groups as appropriate. The Chi-square or Fisher’s exact test will be used to compare the frequencies of categorical variables. Pearson’s correlation will be used to analyse the relationship between C-L classification and the number of attempts until successful intubation. All data will be analyzed using SPSS version 20.0 software (IBM Corp., Armonk, NY, USA).

**11. Ethics and regulation**

1) This study protocol conformed to the ethical guidelines of the 1975 Helsinki Declaration and International Conference on Harmonisation of Technical Requirements of Pharmaceuticals for Human Use (ICH) Note for Guidance on Good Clinical Practice (ICH, Topic E6, 1995)

2) This study was approved by the Institutional Review Board of Samsung Medical Center.

3) Compensation

There is no financial compensation for patients who participate in this study.

**References**

1. Campos JH. Which device should be considered the best for lung isolation: double-lumen endotracheal tube versus bronchial blockers. Curr Opin Anaesthesiol 2007;20:27-31.

2. David Michael Stout et al. Correlation of endotracheal tube size with sore throat and hoarseness following general anesthesia. Anesthesiology 1987;67:419-21.

3. The Intubation Difficulty Scale. Anesthesiology 1997;87-1290-7.

4. Zhong T. et al. Sore throat or hoarse voice with bronchial blockers or double-lumen tubes for lung isolation: a randomised, prospective trial. Anaesth Intensive Care 2009;37:441-6.

5. Knoll H et al. Airway injuries after one-lung ventilation: a comparison between double-lumen tube and endobronchial blocker: a randomized, prospective, controlled trial. Anesthesiology 2006;105:471-7

6. McHardy FE et al. Postoperative sore throat: cause, prevention and treatment. Anaesthesia 1999;54:444-53.

7. Woo P. et al.  Diagnostic-value of stroboscopic examination in hoarse patients. Journal of Voice 1991;5:231-8.

8. Capan LM et al.  Succinylcholine-induced postoperative sore throat. Anesthesiology 1983;59:202-6.

9. Simon Wasem et al. Comparison of the Airtraq and the Macintosh laryngoscope for double-lumen tube intubation. EJA 2013; 30:180-186.

10. T. Russell et al. A randomised controlled trial comparing the GlideScope and the Macintosh laryngoscope for double-lumen endobronchial intubation. Anaesthesia 2013; 68:1253-1258.

**Case Report Form Case No.**

**The fast and easy way for double-lumen tube intubation: Individual angle-modification:**

**A randomized clinical trial**

Study date: Date of randomization:

Log No.:

Study group: Modified angle ( ) / Conventional angle ( )

Op name:

Comments:

| Age | | | |  | | | | | | | | | Sex | | | | | | | | | M / F | | |
| --- | --- | --- | --- | --- | --- | --- | --- | --- | --- | --- | --- | --- | --- | --- | --- | --- | --- | --- | --- | --- | --- | --- | --- | --- |
| ASA class | | | |  | | | | | | | | | Ht/ Wt/ BMI | | | | | | | | |  | | |
| **Airway characteristics** | | | | | | | | | | | | | | | | | | | | | | | | |
| Inter-incisor distance | | | | | | cm | | | | | Passive mouth open | | | | | | | | | | | | cm | |
| Mallampati grade | | | | | | I / II / III / IV | | | | | Thyromental distance | | | | | | | | | | | | cm | |
| Cormack Lehane | | | | | | I / II / III / IV | | | | | Cormack Lehane  (with BURP) | | | | | | | | | | | | I / II / III / IV | |
| **Hemodynamic data during intubation** | | | | | | | | | | | | | | | | | | | | | | | | |
| 1^st^ Attempt | | | | | | | | | | | | | | | | | | | | | | | | |
|  | | | baseline | | | | | 1min | | | | | | | 2min | | | | 3min | | | | | 5min |
| HR | | |  | | | | |  | | | | | | |  | | | |  | | | | |  |
| Systolic BP | | |  | | | | |  | | | | | | |  | | | |  | | | | |  |
| Any drugs | | |  | | | | |  | | | | | | |  | | | |  | | | | |  |
| 2^nd^ Attempt | | | | | | | | | | | | | | | | | | | | | | | | |
|  | | | baseline | | | | | 1min | | | | | | | 2min | | | | 3min | | | | | 5min |
| HR | | |  | | | | |  | | | | | | |  | | | |  | | | | |  |
| Systolic BP | | |  | | | | |  | | | | | | |  | | | |  | | | | |  |
| Any drugs | | |  | | | | |  | | | | | | |  | | | |  | | | | |  |
| 3^rd^ Attempt | | | | | | | | | | | | | | | | | | | | | | | | |
|  | | | baseline | | | | | 1min | | | | | | | 2min | | | | 3min | | | | | 5min |
| HR | | |  | | | | |  | | | | | | |  | | | |  | | | | |  |
| Systolic BP | | |  | | | | |  | | | | | | |  | | | |  | | | | |  |
| Any drugs | | |  | | | | |  | | | | | | |  | | | |  | | | | |  |
| **Intubation data** | | | | | | | | | | | | | | | | | | | | | | | | |
| Tube size | | | | | 32 / 35 / 37 / 39 / 41 | | | | | | | | | | | | | | | | | | | |
| 1st Attempt | | | | | | | | | | | | | | | | | | | | | | | | |
| Time of start | | | | |  | | | | | | | | | Time of end | | | | | |  | | | | |
| Intubation success | | | | | Success / Fail | | | | | | | | | BURP | | | | | | Y / N | | | | |
| CL grade | | | | | I / II / III / IV | | | | | | | | | | | With BURP: I / II / III / IV | | | | | | | | |
| Any oropharyngeal trauma | | | | | | | | | | Y / N ( ) | | | | | | | | | | | | | | |
| Blood at laryngoscope blade | | | | | | | | | | Y / N ( ) | | | | | | | | | | | | | | |
| 2nd Attempt | | | | | | | | | | | | | | | | | | | | | | | | |
| Time of start | | | | |  | | | | | | | | | Time of end | | | | | |  | | | | |
| Intubation success | | | | | Success / Fail | | | | | | | | | BURP | | | | | | Y / N | | | | |
| CL grade | | | | | I / II / III / IV | | | | | | | | | | | With BURP: I / II / III / IV | | | | | | | | |
| Any oropharyngeal trauma | | | | | | | | | | Y / N ( ) | | | | | | | | | | | | | | |
| Blood at laryngoscope blade | | | | | | | | | | Y / N ( ) | | | | | | | | | | | | | | |
| 3rd Attempt | | | | | | | | | | | | | | | | | | | | | | | | |
| Time of start | | | | |  | | | | | | | | | Time of end | | | | | |  | | | | |
| Intubation success | | | | | Success / Fail | | | | | | | | | BURP | | | | | | Y / N | | | | |
| CL grade | | | | | I / II / III / IV | | | | | | | | | | | With BURP: I / II / III / IV | | | | | | | | |
| Any oropharyngeal trauma | | | | | | | | | | Y / N ( ) | | | | | | | | | | | | | | |
| Blood at laryngoscope blade | | | | | | | | | | Y / N ( ) | | | | | | | | | | | | | | |
| **Postoperative data** | | | | | | | | | | | | | | | | | | | | | | | | |
|  | | | | | | | Postoperative 30 min | | | | | | | | | | Postoperative 24 hr | | | | | | | |
| Sore throat | | | | | | |  | | | | | | | | | |  | | | | | | | |
| Hoarseness | | | | | | |  | | | | | | | | | |  | | | | | | | |
| 0 : none, 1: mild, 2: moderate, 3: severe | | | | | | | | | | | | | | | | | | | | | | | | |
|  | **임상 시험 책임자 서명** | | | | | | | | | | | | | | | | | | | | | | | |
|  | 본인은 위의 피험자로부터 적절한 방법에 따라 문서 동의를 얻었으며, 본 증례기록지에 기록된 모든 내용이 위의 피험자로부터 정확히 얻어진 결과임을 확인합니다.  본인 또는 본인이 위임하는 자는,   1. 본 증례 기록지의 내용을 모두 검토하였으며, 2. 그 내용이 정확하고 3. 기록된 날짜에 시행된 검사 또는 측정의 결과임을 확인합니다. | | | | | | | | | | | | | | | | | | | | | | | |
|  | 시험자 성명 |  | | | | | | | 서명 | | |  | | | | | | 날짜 | | | 년 월 일 | | | |
